# A Comprehensive Analysis of Choroideremia: From Genetic Characterization to Clinical Practice

**DOI:** 10.1371/journal.pone.0151943

**Published:** 2016-04-12

**Authors:** Rocio Sanchez-Alcudia, Maria Garcia-Hoyos, Miguel Angel Lopez-Martinez, Noelia Sanchez-Bolivar, Olga Zurita, Ascension Gimenez, Cristina Villaverde, Luciana Rodrigues-Jacy da Silva, Marta Corton, Raquel Perez-Carro, Simona Torriano, Vasiliki Kalatzis, Carlo Rivolta, Almudena Avila-Fernandez, Isabel Lorda, Maria J. Trujillo-Tiebas, Blanca Garcia-Sandoval, Maria Isabel Lopez-Molina, Fiona Blanco-Kelly, Rosa Riveiro-Alvarez, Carmen Ayuso

**Affiliations:** 1 Department of Genetics, Instituto de Investigacion Sanitaria-Fundacion Jimenez Diaz (IIS-FJD), Madrid, Spain; 2 Centro de Investigacion Biomedica en Red de Enfermedades Raras (CIBERER), ISCIII, Madrid, Spain; 3 Inserm U1051, Institute for Neurosciences of Montpellier, Montpellier, France; 4 Department of Medical Genetics, University of Lausanne, Lausanne, Switzerland; 5 Department of Ophthalmology, IIS-Fundación Jiménez Díaz University Hospital, Madrid, Spain; Innsbruck Medical University, AUSTRIA

## Abstract

Choroideremia (CHM) is a rare X-linked disease leading to progressive retinal degeneration resulting in blindness. The disorder is caused by mutations in the *CHM* gene encoding REP-1 protein, an essential component of the Rab geranylgeranyltransferase (GGTase) complex. In the present study, we evaluated a multi-technique analysis algorithm to describe the mutational spectrum identified in a large cohort of cases and further correlate *CHM* variants with phenotypic characteristics and biochemical defects of choroideremia patients. Molecular genetic testing led to the characterization of 36 out of 45 unrelated CHM families (80%), allowing the clinical reclassification of four CHM families. Haplotype reconstruction showed independent origins for the recurrent p.Arg293* and p.Lys178Argfs*5 mutations, suggesting the presence of hotspots in *CHM*, as well as the identification of two different unrelated events involving exon 9 deletion. No certain genotype-phenotype correlation could be established. Furthermore, all the patients´ fibroblasts analyzed presented significantly increased levels of unprenylated Rabs proteins compared to control cells; however, this was not related to the genotype. This research demonstrates the major potential of the algorithm proposed for diagnosis. Our data enhance the importance of establish a differential diagnosis with other retinal dystrophies, supporting the idea of an underestimated prevalence of choroideremia. Moreover, they suggested that the severity of the disorder cannot be exclusively explained by the genotype.

## Introduction

Choroideremia (CHM; MIM 30100) is an X-linked retinal dystrophy affecting 1 in 50.000 individuals [1]. It is caused by mutations in the *CHM* gene, encoding the Rab escort protein 1 (REP-1). Typically, males with CHM develop progressive peripheral visual field loss beginning with night blindness in their teenage years, leading to complete blindness later in life. Female carriers are generally asymptomatic, although mild signs as pigmentary changes can be observed. These symptoms are very similar to those of other retinal dystrophies, including RP, so a proper differential diagnosis needs to be carried out.

The *CHM* gene is located on chromosome X at position Xq21.2. Its mRNA (NM_000390) spans 15 exons and is 5442 bp long. The open reading frame is 1962 bp and encodes the ubiquitously expressed protein REP-1, composed of 653 aminoacids. REP-1 is an essential component of the Rab geranylgeranyltransferase (GGTase) complex [2]. It binds unprenylated Rab proteins and escorts them to Rab-GGTase for the transfer of geranyl-geranyl groups, necessary for membrane association and target-protein recognition [3–5]. In the absence of this post-translational modification, Rabs proteins cannot participate in pathways of intracellular vesicular transport. Since REP-1 is particularly crucial for the function of the retinal pigment epithelium and photoreceptors, its depletion leads to cell degeneration and subsequent choroidal tissue loss.

Mutational spectrum characterized in *CHM* revealed deletions, insertions, duplications, translocations, nonsense, splice-site, frameshift and missense mutations (http://www.lovd.nl/CHM). Genotype-phenotype correlations remain unclear, limited information is available on the early phenotypic manifestations in molecularly characterized patients; and the precise disease mechanisms are still unknown. The aim of this study is to describe the mutational spectrum in our cohort of cases as well as the multi-technique diagnostic pipeline to follow up in patients with CHM. We also aimed at further correlating *CHM* variants with phenotypic characteristics and biochemical defects of choroideremia patients.

## Material and Methods

### Ascertainment of patients

All patients´ samples proceed from the Biobank of University Hospital Fundacion Jimenez Diaz. We have analyzed forty-one unrelated families, mostly from Iberian population, with an initial clinical diagnosis of CHM. Four additional families reclassified were also studied. Written informed consent was obtained from patients participating in this study, and the research protocols were approved by the bioethical committee and were in accordance with the Declaration of Helsinki and further reviews (Fortaleza, 2013). Diagnosis of choroideremia was determined in patients with suspected or not excluded X linked inheritance and with night blindness, peripheral vision loss, characteristic fundus appearance with patchy areas of chorioretinal degeneration generally begin in the mid-periphery of the fundus and reduced scotopic electroretinogram response. The ophthalmic tests performed included best-corrected visual acuity (BCVA), intrinsic and extrinsic motility, anterior pole and fundus examination, static perimetry, color testing, and Ganzfeld electroretinography according to the International Society for Clinical Electrophysiology of Vision (ISCEV) Standards. All patients´ DNA samples were extracted as previously described [6].

### Direct sequencing of the *CHM* gene

All exons of the *CHM* gene, along with adjacent intronic sequences, were amplified from genomic DNA by PCR using primers and conditions previously described [6].

### Copy number variation (CNV) analysis

CNV analysis was initially performed using quantitative fluorescent PCR (qF-PCR) and/or Multiplex Ligation dependent Probe Amplification (MLPA). For qF-PCR, multiplex amplification was performed as follows: for each sample two reactions, set A and set B containing primers to amplify even- or odd-numbered exons, respectively, were set up. In each of the sets, control primers along chromosome X (control-A and E), as well as in the regions adjacent to the *CHM* gene that are involved in some rearrangements, previously detected in CHM patients (control-B, C and D), were included. In all cases, forward primers were labelled with FAM fluorochrome. Amplification products were separated by electrophoresis (ABI Prism 3130; Applied Biosystems) and analyzed with a software package (GeneMapper v3.5; Applied Biosystems). For MLPA, 250 ng of genomic DNA were used as starting material with the SALSA P366-A1 CHM-RP2-RPGR MLPA kit available from MRC Holland, Amsterdam (www.mrc-holland.com) following the manufacturer´s instructions. To characterize complete *CHM* deletions, we used Agilent Human Genome CGH Microarray 244K (with median spacing of 8.9kb) and Agilent SurePrint G3 CGH+SNP 2x400k Microarray kits (containing 292,097 CGH probes and 119,091 SNP probes with median spacing of 7.2kb). To characterize partial *CHM* deletions we used the custom aCGH 8X60k using Agilent SurePrint G3 CGH, which represents an average distribution of one probe per 150 bp in the *CHM* gene. Briefly, patients and sex-matched control samples were labeled after the digestion with Cy3-dUTP and Cy5-dUTP fluorochromes using the Sure Tag DNA Labeling Kit (Agilent Technologies). The labeled products were purified, hybridized and washed according to Agilent protocols. The results of 8X60k, 2X400k and 244k kits were analyzed by Agilent CytoGenomics v.2.7 and Agilent DNA Analytics 4.0 softwares, using default analysis methods—CGH v2 with the ADM-2 aberration and ADM1 aberration algorithms, respectively.

### Cytogenetic studies

Karyotype and cytogenetic studies were performed as previously described[7].

### Haplotype analysis

To determine the X-linked inheritance pattern and the implication of *CHM*, haplotype analysis were performed using microsatellite markers flanking the *CHM* gene (DXS1002-DXS8076) and three intragenic polymorphic markers (one single nucleotide polymorphism (SNP) in exon 5, one variable number tandem repeat (VNTR) in intron 9 and one short tandem repeat (STR) in intron 14). To determine whether several families shared a common ancestor, haplotypes were generated by using two intragenic polymorphic markers (one SNP in exon 5 and one STR in intron 14) and four additional microsatellite markers closely flanking the *CHM* gene (TEL-DXS990-DXS1002-REP1-DXS8076-DXS986). Direct sequencing was performed for the genotyping analysis of the SNP in exon 5. The rest of the markers used were separately amplified. Each forward primer was fluorescence labelled and amplification products were separated by electrophoresis (ABI Prism 3130; Applied Biosystems) and analyzed with a software package (GeneMapper v3.5; Applied Biosystems). For haplotype reconstruction, an informatics program was utilized (Cyrillic ver. 2.1; Cyrllic Software, Wallingford, UK).

### Next-Generation Sequencing (NGS)

For Whole Exome Sequencing (WES), genomic DNA, library and sequencing were performed as previously described [8]. A custom NGS panel of 37 genes involved in retinal dystrophies, including the *CHM* gene, was developed as follows: a total of 588 target regions (1,472 amplicons) were entered into DesignStudio software (Illumina). Once the oligonucleotide probes were synthesized, libraries were constructed and indexed by PCR using common primers from the TruSeq Amplicon Index Kit (Illumina). Finally, libraries were normalized and pooled, prior to sequencing on a NextSeq500 system (Illumina).

### Fibroblast cultures

Skin biopsies of patients carrying a deletion of the *CHM* gene, a UAA nonsense mutation and a UGA nonsense mutation were performed following informed consent at the Centre of Reference for Genetic Sensory Disorders (CHRU Montpellier, France). The biopsy of the patient carrying a deletion of exon 8 performed at the same centre was previously reported [9]. The skin biopsy of the patient carrying a UAG nonsense mutation was performed at the IIS-Fundacion Jimenez Diaz (Madrid, Spain). The skin biopsies and emerging fibroblasts were cultured in AmnioMAX C100 basal media with L-glutamine (Invitrogen, Life Technologies, Saint Aubin, France) containing 10% decomplemented FCS (Lonza, Verviers, Belgium), 1% penicillin-streptomycin-amphotericin B (Lonza) and 2% AmnioMax-C100 supplement (Invitrogen, Life Technologies) at 37°C under 5% CO2 as described [9]. This work was performed under the biomedical research authorization number 2014-A00549-38.

### *In vitro* prenylation assay

At confluence, the patient’s fibroblasts cultured in a 6-well plate, were washed in cold PBS, scraped in PBS containing antiproteases and pelleted at 3000g for 5 min. The pellet was resuspended in cold, freshly prepared degassed prenylation/lysis buffer and *in vitro* prenylation was performed as described [9]. Western blot detection was performed using enhanced chemiluminescence system ECL (Life Technologies). The amount of biotinylated Rab proteins was then quantified by scanning densitometry using the software Image J and expressed as a function of the β-actin signal. Experiments were performed in triplicate. Due to small sample sizes, 2x2 comparisons were performed using a non-parametric Mann-Whitney test.

## Results

A total of 41 families with initial clinical diagnosis of CHM were included in our molecular diagnostic pipeline. Four additional reclassified families were also studied. The study resulted in the identification of 36 families with mutations in *CHM*, including 46 affected individuals.

### CNV analysis

We identified three different families with gross deletions encompassing the entire *CHM* gene. All of them were further delimited by aCGH. In two of the families (RP-0747 and RP-1226) a novel 1.9 Mb deletion and a previously described 1.5 Mb deletion [6], were found at chromosome region arr [18] Xq21.2q21.31 (84692099–86623284) x0 and arr [18] Xq21.2q21.31 (84879062–86393588) x0, respectively, encompassing both the *CHM* and *DACH2* genes. In the other family, RP-1959, a firstly demarcated 455.6 kb deletion at chromosome region arr [19] Xq21.2 (84847610–85303270) x0 exclusively encompassing the entire *CHM* gene was found.

**Fig 1:**
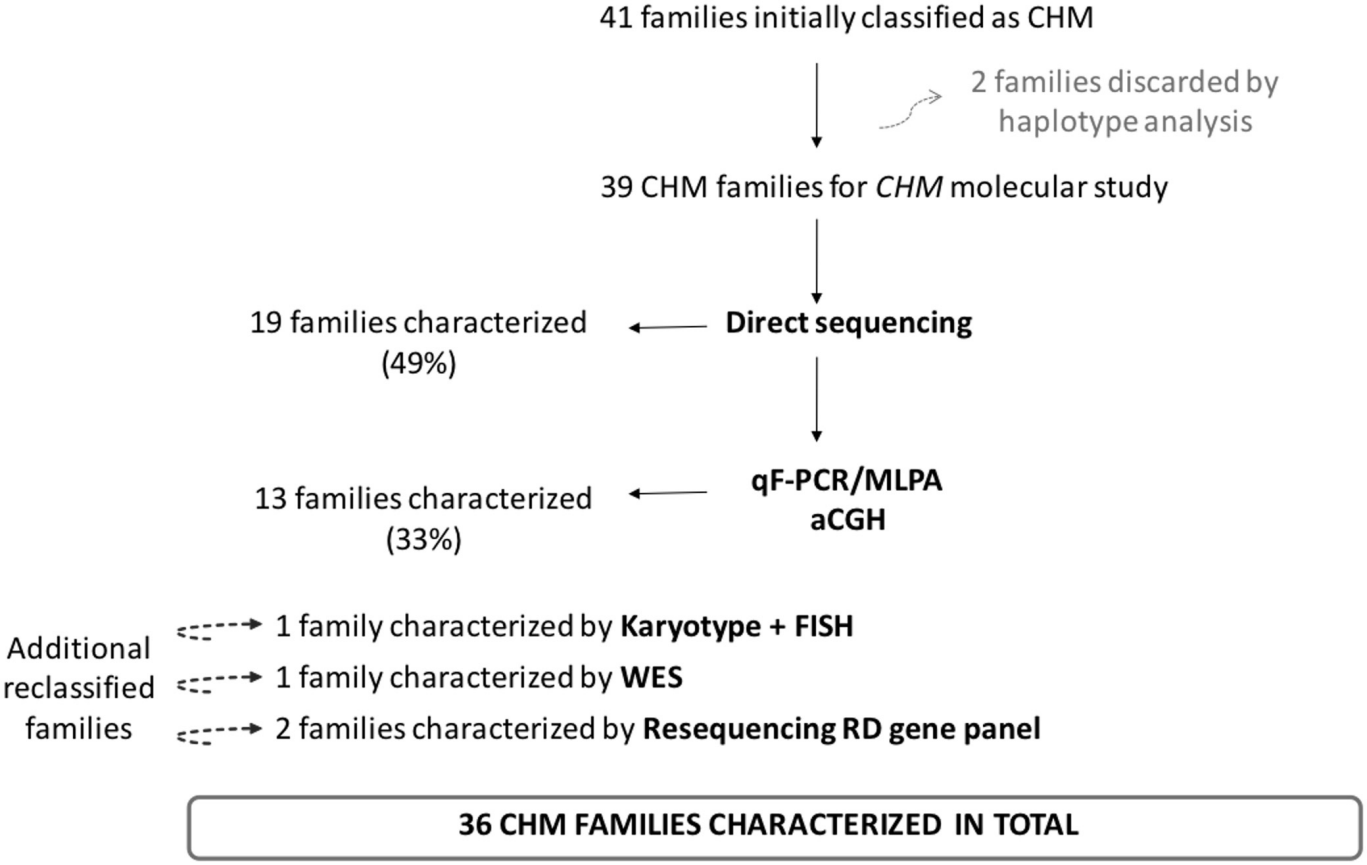
Molecular strategy followed up for the diagnosis of CHM families.

In the majority of the cases, QF-PCR was the preferred technique when partial *CHM* deletions were suspected. Ten families with different partial deletions involving the promoter region and/or various exons were found (Fig 1 and Table 1). Among them, the deletion of exon 9 was the most common, being identified in four families (three Spanish families RP-1310, RP-1560 and RP-2128 and a Portuguese family RP-0779). QF-PCR, in combination with MLPA, was used in all of them. This combination of tools, in addition to confirming the presence of the deletion, helped to precisely delimit it. In all the cases, except in the RP-0779 family, the deletion was delimited to exon 9 and the adjacent intronic region (less than 439 nucleotides, as the exon 9 probe located at this position was present). In the RP-0779 family, we demarcated by further aCGH a deletion of a minimum of 11,149 bp involving exon 9 and most of intron 9. Two additional deletions were also delimited by aCHG. Deletion of exon 2 in the RP-0317 family was demarcated leading to a genomic deletion of 1993 bp at chromosome region arr [18] Xq21.2 (85168495–85170487) x0. A genomic deletion of 48.6kb, arr [19] Xq21.2 (85233612–85282246) x0, encompassing both exon 3 and 4, was delimited in the RP-0918 family.

**Table 1:** Mutational spectrum of CHM characterized families.

Mutation	Effect	Methods	Origin	Described/Novel^a^	n	Relative frequency (%)^b^
**Translocation**						
46,X,t (X;4)(q21:p16)	*de novo* translocation X;4 [t X;4]	Karyotype+FISH	Spain	Described in our cohort[7]	1	3
**Genomic deletion**						
c.-29-*3450del1931185	Complete *CHM* and *DACH2* deletion	qF-PCR+aCGH	Spain	This study	1	3
c.-29-*3450del1514526	Complete *CHM* and *DACH2* deletion	qF-PCR+aCGH	Spain	Described in our cohort[6]	1	3
c.-29-*3450del445660	Complete *CHM* deletion	qF-PCR+aCGH	Spain	This study	1	3
c.-29-?_49+?del(no longer 24.4Kb)	Promoter to intron 1 deletion	Direct sequencing+ aCGH	Spain	Firstly demarcated in this study	1	3
c.-29-?_116+?del	Promoter to exon 2 deletion	Direct sequencing+ aCGH	Spain	Described	1	3
c.50-116del1992	Exon 2 deletion	RNA studies+aCGH	Spain	Firstly demarcated in this study	1	3
c.189+34_*3450+?del	Exon 4 to exon 15 deletion	qF-PCR+MLPA	Spain	Firstly demarcated in this study	1	3
c.117-314del48634	Exon 3 and exon 4 deletion	RNA studies+aCGH	Spain	Firstly demarcated in this study	1	3
c.703-?_940+?	Exon 6 and exon 7 deletion	qF-PCR	Unknown	Described	1	3
c.1167-1244del11149	Exon 9 deletion	qF-PCR+MLPA	Portugal	Firstly demarcated in this study	1	3
c.1167-?_1244+?del	Exon 9 deletion	qF-PCR+MLPA	Spain	Described	3	9
**Splicing**						
c.189+1G>A	Exon 3 skipping	Direct sequencing/NGS panel	Spain	Described	2	6
c.1167-1G>T	Exon 9 skipping	NGS panel	Spain	This study	1	3
c.1167-2A>G	Exon 9 skipping	Direct sequencing	Spain	This study	1	3
**Frameshift**						
c.641_642delGA	p.Arg214Asnfs*8	Direct sequencing	Spain	Described in our cohort[6]	1	3
c.525_526delAG	p.Lys178Argfs*5	Direct sequencing	1 Portugal; 1 Spain	Described	2	6
c.862dupA	p.Thr288Asnfs*19	WES	Spain	Described in our cohort[6]	1	3
**Nonsense**						
c.116C>A	p.Ser39*	Direct sequencing	Spain	Described	1	3
c.141G>A	p.Trp47*	Direct sequencing	Spain	Described	2	6
c.256C>T	p.Gln76*	Direct sequencing	Spain	Described	1	3
c.339T>G	p.Tyr103*	Direct sequencing	Spain	Described	1	3
c.745C>T	p.Arg239*	Direct sequencing	Spain	Described	1	3
c.877C>T	p.Arg293*	Direct sequencing	1 Portugal; 1 Poland;2 Spain	Described	4	12
c.1048C>A	p.Ser340*	Direct sequencing	Spain	Described in our cohort [10]	1	3
c.1272_1273invTC	p.Gln425*	Direct sequencing	Spain	This study	1	3
c.1471G>T	p.Glu491*	Direct sequencing	Spain	Described	1	3
c.1703T>A	p.Leu568*	Direct sequencing	Spain	This study	1	3
**Missense**						
c.49G>T	p.Gly17Cys	Direct sequencing	Belgium	This study	1	3

^**a**^ Present on Human Gene Mutation Database (HGMD) or Locus specific database (LSDB).

^**b**^ Calculated relative to 36 CHM families characterized in the laboratory.

### Mutational spectrum and molecular genetic testing

In families for which affected members were available for investigation, haplotype analysis was performed to confirm the segregation of *CHM* with the disease. Following this approach, two families were discarded for further *CHM* molecular studies. For the remainder, direct sequencing of the exons and adjacent introns of the gene detected *CHM* mutations in 19 families, corresponding to a 49% detection rate. Specifically, we identified 3 different splicing mutations, 3 frameshift, 9 nonsense and one missense mutation. For the rest of the families, when direct sequencing failed or was insufficient to accurately detect *CHM* mutations, alternative techniques such as qF-PCR, sometimes in combination with MLPA, were used to detect copy number variations (CNVs). This strategy, resulting in a 33% detection rate, allowed us to characterize 13 families and to identify 11 complete or partial deletions involving multiple or single exons (Fig 1 and Table 1). However, when complete deletion of the gene was suspected, as occurred in three of the families, aCGH was the technique of choice to accurately identify the exact genomic region and the possible involvement of any adjacent gene. Four additional families were clinically reclassified to CHM by additional studies. By karyotype analysis, a translocation between chromosomes X and 4, disrupting *CHM* [7] was found in one family. Next-Generation Sequencing allows to reclassified 3 additional families, one by WES [8] and two families by resequencing RD gene panel (Fig 1 and Table 1). In some cases, RNA and immunoblot analyses were used as a complementary strategy to predict the effect of the variant in the mRNA or in the protein [6].

Complete or partial deletions of *CHM*, found in 13 families (36%) in total, as well as nonsense (13 families; 36%), were the most frequent mutations identified in our cohort. Among the deletions, exon 9 was the most recurrent one (3 families; 8%). From the families carrying nonsense mutations, c.877C>T (p.Arg293*) was the most frequent one, having been found in 4 families (11%) from diverse origins: Spain, Portugal and Poland. The frameshift c.525_526delAG (p.Lys178Argfs*5) mutation was found both in one Spanish and in one Portuguese family (Table 1). The nucleotide substitution c.49G>T, reported for the first time in this study, was not present in our in-house exome variant database nor in any public database. It was predicted to produce the substitution p.Gly17Cys, indicated as putatively pathogenic by the SIFT, Poplyphen-2 and Mutation Taster softwares. However, since it is located in the last nucleotide of exon 1, it could also affect the normal splicing of the *CHM* primary transcript. Indeed, according to HSF tool, it decreases the score of the 5´splice site of intron 1 (from 86.34 to 75.47) possibly also by interfering with the recognition of splicing signals by U1snRNP.

### Hot spots in *CHM*

**Fig 2:**
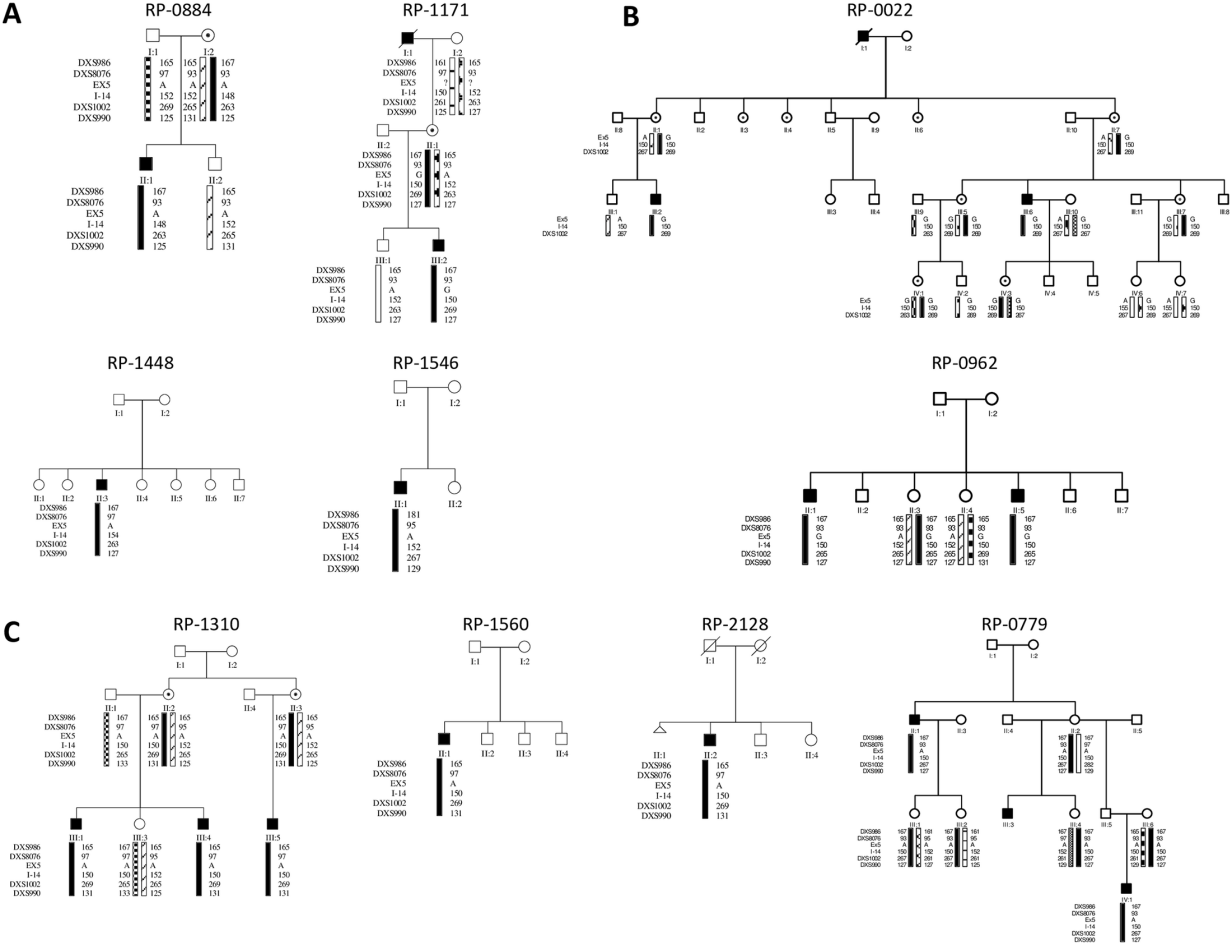
Haplotypes from families presenting the recurrent *CHM* mutations. Identified pedigrees carrying the exon 9 deletion **(A)**, the p.Arg293* **(B)** and the p.Lys178Argfs*5 **(C)** mutations are shown. For exon 9 deletion, haplotypes analysis demonstrated identity by descent in the Spanish families RP-1310, RP-1560 and RP-2128 but independent origin for the Portuguese family RP-0779, defined by the alleles located along the black bar. For the p.Arg293* and p.Lys178Argfs*5 mutations, haplotypes indicates an independent origin for both variants defined by the alleles located along the black bar.

Both common ancestors and mutational hotspots could be responsible for the presence of recurrent mutations in unrelated individuals. To gain insights into this matter, we performed the amplification of microsatellite markers, spanning over 13.6 cM between the DXS990 and DXS986 markers and including two intragenic markers, in the affected members of the families carrying recurrent mutations (Fig 2).

As mentioned, exon 9 deletion was found in three Spanish families (RP-1310, RP-1560 and RP-2128). They did not report being related, but they were from geographically proximal areas. Following haplotypes analysis, a founder effect for these families was suggested. However, for the Portuguese family RP-0779 carrying a larger deletion of exon 9, identity by descent was discarded (Fig 2A).

Non-shared haplotypes were found in the affected members of families (RP-0884, RP-1171, RP-1448 and RP-1546) carrying the c.877C>T (p.Arg293*) mutation, occurring at a CpG dinucleotide. The same was true for the geographically unrelated families (RP-0022 and RP-0962) carrying the c.525_526delAG (p.Lys178Argfs*5) mutation (Fig 2B and 2C).

### Genotype-phenotype-genotype correlation

The summary of clinical and genetic data is presented in Table 2. Night blindness is one of the early symptoms reported with a very wide range between individuals (18±12 years old). However, three of the patients presented with nyctalopia in the third or after the fourth decade of life. Loss of peripheral visual field appeared mostly in the second decade of life. There was a large range of variations in the visual acuity parameters between the affected individuals, even between both eyes in the same individual, and therefore this parameter does not seem to represent a reliable feature of the disease. Electroretinogram was abolished in most of the patients around their fourth decade of life. Subcapsular cataracts were only found in 2 out of 36 of the families, indicating that they are not common signs in CHM.

**Table 2:** Clinical findings identified in affected individuals carrying mutations in the *CHM* gene.

Family	Mutation	Subject	First symptoms and course	Age of ophthalmic evaluation (y)	BVCA OD/OS	Fundus aspect	Visual Field	ERG	Additional findings
**RP-0288**	Translocation X;4 [t X;4]	Proband	NB (5y), field constriction (23y) and loss of VA (34y)	ND	0.5/0.5	Choroidal atrophy	Loss of peripheral VF remaining central island	Reduced	Cataracts
**RP-1226**	Complete *CHM* and *DACH2* deletion	Proband	NB (3y)	5	0.8/0.8	Pigment clumping	ND	ND	
		Sibling	NB (8y)	10	0.8/0.8	Pigment clumping	Loss of peripheral VF remaining central island	ND	
		Sibling	NB (10y)	12	1/1	Pigment clumping	Loss of peripheral VF remaining central island	Reduced	
**RP-0747**	Complete *CHM* and *DACH2* deletion	Proband	NB (11y), field constriction (11y)	ND	1/1	Peripheral choroidal atrophy and focal atrophy of the RPE	Loss of peripheral VF remaining central island	Reduced	
**RP-1959**	Complete *CHM* deletion	Proband	NB (16y)	ND	0.8/0.35	Choroidal atrophy and pigment clumping	Loss of peripheral VF	ND	
**RP-1144**	Promoter to intron 1 deletion	Proband	NB (17y), field constriction (18y) and loss of VA (50y)	63	0.3/0.1	Generalized choroideal atrophy	Loss of peripheral VF remaining central island (>5°)	NR	
		Grandson	NB (4y)	4	0.3/0.1	Pigment clumping		NR	
**RP-1276**	Promoter to exon 2 deletion	Proband	ND	37	>0.1/>0.1	Pigment clumping and areas of choriocapillaries loss	Loss of peripheral VF remaining central island (5°)	NR	
**RP-0317**	Exon 2 deletion	Proband	NB (23y), field constriction (23y) and loss of VA (33y)	3	0.25/0.5	Peripheral choroidal atrophy and focal atrophy of the RPE	Absolute scotoma	NR	Macular oil spill OD (33 y)
		Sibling	NB (21y), field constriction (26y) and loss of VA (17y)	40	0.3/0.8	Generalized choroideal atrophy. Pigment clumping and areas of choriocapillaries loss	Loss of peripheral VF remaining central island	NR	
**RP-0918**	Exon 3 and exon 4 deletion	Proband	NB (64y), field constriction (64y)	66	ND	Peripheral choroidal atrophy and focal atrophy of the RPE	Affected visual field	ND	
**RP-2149**	Exon 4 to exon 15 deletion	Proband	NB (27y)	ND	0.01/LP	Generalized choroideal atrophy. Pigment clumping and areas of choriocapillaries loss	ND	Reduced RE, NR LE	
**RP-1809**	Exon 6 and exon 7 deletion	Proband	ND	ND	ND	ND	ND	NR	
**RP-0779**	Exon 9 deletion	Proband	ND	ND	ND	ND	ND	ND	
**RP-1310**	Exon 9 deletion	Proband	NB (4y)	ND	0.5/0.125	Pigment clumping and areas of choriocapillaries loss	Loss of peripheral VF remaining central island (5–10°)	Reduced	
**RP-1560**	Exon 9 deletion	Proband	NB (8y)	ND	ND	ND	Loss of peripheral VF	ND	
**RP-2128**	Exon 9 deletion	Proband	NB (30y), field constriction (30y) and loss of VA (30y)	ND	0.1/0.3	Diffuse choroidal atrophy	Loss of peripheral VF remaining central island (10°)	ND	
**RP-0022**	p.Lys178Argfs*5	Proband	NB (8y), field constriction (8y) and loss of VA (14y)	43	0.1/0.1	Peripheral choroidal atrophy and focal atrophy of the RPE	Loss of peripheral VF remaining central island	NR	Myopia. Hypoacusia
		Cousin	NB (26y), field constriction (26y) and loss of VA (26y)	ND	ND	ND	ND	ND	Hypermetropia
**RP-0962**	p.Lys178Argfs*5	Proband	ND	ND	ND	ND	ND	ND	
**RP-0889**	p.Arg214Asnfs*8	Proband	NB (28y), field constriction (28y)	28	0.8/CF	ND	Absolute scotoma	NR	
**RP-1164**	p.Thr288Asnfs*19	Proband	NB (18y), field constriction (18y)	28	1/1	Peripheral choroidal atrophy and focal atrophy of the RPE	Loss of peripheral VF remaining central island	Reduced	
		Sibling	NB (28y), field constriction (28y)	28	ND	ND	ND	ND	
**RP-0729**	c.189+1G>A	Proband	NB (26y), field constriction (28y) and loss of VA (35y)	36	0.3/0.4	Pigment clumping with macular atrophy	Loss of peripheral VF remaining central island	NR	
**MD-0495**	c.189+1G>A	Proband	NB (25y), field constriction (24y) and loss of VA	24	0.05/0.05	Atrophy of the RPE	Loss of peripheral VF (>10°)	NR	
**RP-1500**	c.1168-2A>G	Proband	NB (10y), field constriction (20y) and loss of VA (32y)	ND	>0.1/>0.1	Peripheral choroidal atrophy and focal atrophy of the RPE with subsequent exposure of choroidal vessels. Pigment clumping	Loss of peripheral VF	ND	
**RP-1995**	c.1167-1G>T	Proband	NB (30y), field constriction (35y) and loss of VA (31y)	30	Legal blindness	Choroidal atrophy	Absolute scotoma (RE); <10° (OS)	ND	
**RP-1224**	p.Ser39*	Proband	NB (30y), field constriction (35y) and loss of VA (35y)	ND	0.8/LP	ND	ND	ND	
**RP-2199**	p.Trp47*	Proband	NB (19y), field constriction (33y) and loss of VA (33y)	36	0.7/0.8	Peripheral choroidal atrophy and focal atrophy of the RPE	Loss of peripheral VF remaining central island	NR	
**RP-1098**	p.Gln76*	Proband	NB (22y), field constriction (21y)	27	Amaurosis (ptisis bulbi)/1	Peripheral choroidal and Bruch´s membrane atrophy with pigment clumping	Loss of peripheral VF remaining central island	NR	
**RP-0797**	p.Tyr103*	Proband	NB (44y), field constriction (40y) and loss of VA (44y)	46	0.4/CF	Generalized choroidal atrophy	Loss of peripheral VF remaining central island	NR	Epilepsy (42y)
**RP-0590**	p.Arg239*	Proband	NB (16y), field constriction (27y)	27	1/1	Pigment clumping and areas of choriocapillaries loss	Loss of peripheral VF remaining central island (10°)	NR	
		Sibling	NB (6y), field constriction (18y)	19	0.6/0.7	Pigment clumping and areas of choriocapillaries loss	Loss of peripheral VF remaining central island (5°)	NR	Cataracts
		Sibling	NB (16y), field constriction (20y)	28	1/1	Pigment clumping and areas of choriocapillaries loss	Loss of peripheral VF remaining central island (5°)	NR	
**RP-0884**	p.Arg293*	Proband	ND	27	ND	ND	ND	ND	
**RP-1171**	p.Arg293*	Proband	NB (8y), field constriction (26y) and loss of VA (15y)	ND	0.6/0.6	ND	Loss of peripheral VF	NR	
**RP-1448**	p.Arg293*	Proband	NB (8y), field constriction (15y) and loss of VA (30y)	39	ND	Peripheral choroidal atrophy and focal atrophy of the RPE	Loss of peripheral VF	NR	
**RP-1546**	p.Arg293*	Proband	NB (13y), field constriction (23y) and loss of VA (23y)	ND	ND	ND	ND	NR	
**RP-0023**	p.Ser340*	Proband	NB (7y), field constriction (17y) and loss of VA (7y)	44	CF/HM	Pigment clumping with macular atrophy	Loss of peripheral VF	NR	
		Uncle	Loss of VA (52y)	ND	0.8/0.9	Peripheral choroidal atrophy and pigment clumping	Loss of peripheral VF remaining central island	ND	
**RP-0411**	p.Gln425*	Proband	NB (20y), field constriction (24y) and loss of VA (20y)	ND	0.1/0.1	Peripheral choroidal atrophy	Loss of peripheral VF remaining central island (10–15°)	NR	
		Sibling	ND	ND	1/1	Peripheral choroidal atrophy	Loss of peripheral VF remaining central island (20–30°)	NR	
**RP-1508**	p.Glu491*	Proband	ND	ND	ND	ND	ND	ND	
**RP-2342**	p. Leu568*	Proband	NB (7y), field constriction (27y) and loss of VA (38y)	7	0.05/0.4	Optic pallor, choroidal atrophy, thin retinal vessels.	Affected visual field	NR	
**RP-1495**	p.Gly17Cys	Proband	ND	ND	ND	ND	ND	ND	

BCVA: best corrected visual acuity; OD: right eye; OS: left eye; ERG: electroretinogram; NB: night blindness; VA: visual acuity; VF: visual field; ND: no data; NR: non recordable; RPE: retinal pigmented epithelium; CF: counting fingers; LP: light perception; HM: hand motion

In our cohort of patients we could not find significant differences between the age of onset, field constriction or loss of VA and the different types of mutations to be able to establish a clear genotype-phenotype correlation. The only observation we noticed was that the group of patients carrying complete deletion of the gene referred an early onset nyctalopia (9.6±4.7 years old), although this data are not statistically significant (p-value = 0.11). Moreover, no differences in the phenotype were observed in the patients carrying deletions involving *CHM*, solely or in conjunction with its adjacent gene *DACH2*, also indicating that this latter gene has probably no role in CHM pathogenesis.

### Functional assay

**Fig 3:**
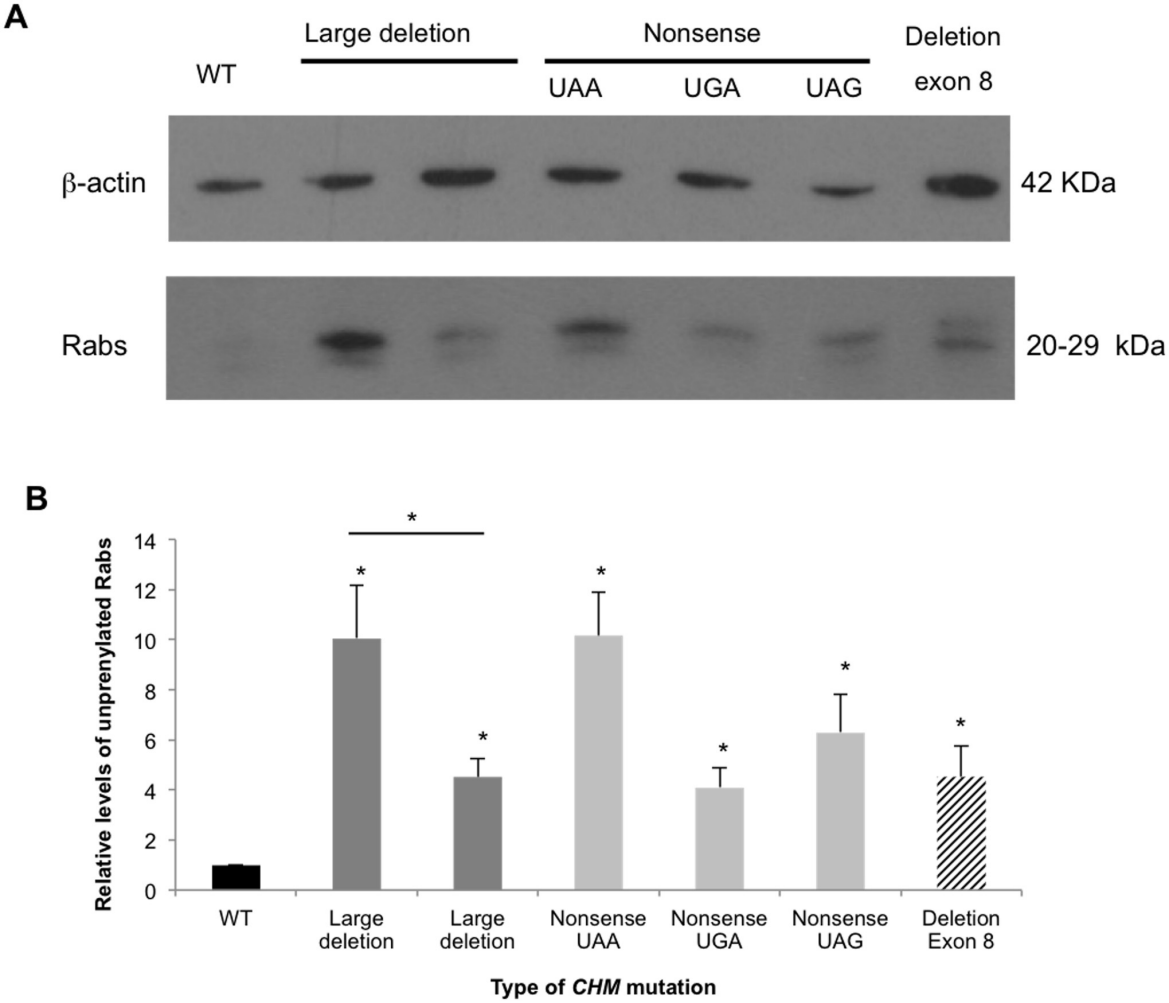
Prenylation status of different *CHM* mutations. **A)** A representative *in vitro* prenylation assay using a biotinylated prenyl donor followed by western blot analysis. A weaker signal of incorporated biotin can be seen for the wild-type (WT) control as compared to the patients’ cells. **B)** Semi-quantification of the pool of biotinylated Rabs (ranging in size from 20 to 29 kDa) after normalization of β-actin loading. The levels in control cells were set to 1. The unprenylated Rab levels were significantly higher than wild-type (black bar) in all the patients’ cells regardless of the type of mutation (p<0.05, asterisks; n = 3). In addition, the Rab levels were significantly different between two brothers carrying the same large deletion (dark grey bars) of the *CHM* gene (p<0.05, asterisk; n = 3).

As there were no correlations at the clinical level, we tested the effect of different mutations at the functional level by assaying whether Rab GTPase prenylation is affected differently depending on the type of *CHM* mutation. To this end, we measured the levels of unprenylated Rabs in the fibroblasts of 6 patients carrying different classes of *CHM* mutations (deletion of the whole gene, nonsense mutations or deletion of a single exon). There was no REP-1 protein detectable in any of the patients’ fibroblasts (data not shown). Consistently, all of the patients’ fibroblasts presented significantly increased levels of unprenylated Rabs compared to control cells (ranging from 4- to 10-fold higher, p<0.05). There were also significant differences in levels between the patients however this was not related to the genotype as the highest levels were present in one of the patients carrying a whole deletion of the gene and in a patient carrying a UAA nonsense mutation in exon 6 (Fig 3). In contrast, the unprenylated Rab levels were lower (2-fold on average) in the patients carrying the two other nonsense mutations (UGA in exon 14 and UAG in exon 4). Interestingly, we noted that the two siblings carrying a whole *CHM* deletion (family RP-1226) presented significantly different levels of unprenylated Rabs (2.5-fold, p<0.05).

## Discussion

Choroideremia is a very genetically heterogeneous disease in which different types of mutations are reported. In this study, we evaluated 41 families initially clinically diagnosed with CHM and 4 additional families re-diagnosed as CHM. We characterized 36 families in total and identified 28 different mutations. Most of the families (31%, 22/36) carried hemizygous nucleotide substitutions, corresponding mostly to nonsense mutations (36%, 13/36), although splicing mutations (11%, 4/36), frameshift (11%, 4/36) and missense (3%, 1/36) variants were also identified. The remainder of the families (13/36) carried genomic deletions involving complete or partial deletion of the gene. As an exceptional case, a previously described *de novo* translocation, [7], was also found (Table 1).

The molecular diagnostic pipeline used in this report resulted in an effective method for the identification of *CHM* defects in different populations. In addition, our data supports the idea of an underestimated prevalence of the disease. Some cases were initially incorrectly diagnosed, due to the similarity of symptoms with other retinal dystrophies, but further characterized as CHM by NGS, consistent with previous reports [11] (Fig 1). Moreover, cases of genomic rearrangements, such as the translocation event, and deep intronic mutations that can occur in *CHM* [7, 12, 13], failed to be detected by conventional techniques. As shown here, NGS, both in the form of re-sequencing gene panels and WES, was successful for the identification pathogenic *CHM* variants. However, to date large deletions or duplications and genomic rearrangements cannot always be effectively identified by this technology [14]. Thus, currently we propose this approach as a first step, when the patient displays a clear CHM phenotype, followed by the study of genomic rearrangements in those negative cases. In addition, NGS is also a very good approach for identifying novel CHM cases amongst previous incorrectly diagnosed cases, thus raising disease prevalence.

To date, a small number of missense mutations were identified in *CHM*. Interestingly, the novel c.46G>T variant presumably leads to the missense p.Gly17Cys substitution. However, it is located in the last nucleotide of exon 1, and therefore could probably affect splicing, as is the case for other *CHM* variants located in exonic sequence [15]. Further studies would be necessary to assess mRNA transcripts resulting from these alleles.

Following haplotype reconstruction, we showed that the recurrent p.Arg293* and p.Lys178Argfs*5 mutations had independent origins in different carriers, therefore suggesting the presence of mutational hotspot in *CHM*. This is particularly true for the p.Arg293* mutation located in exon 7, identified in four independent and unrelated families. This mutation is C to T transition, occurring at a CpG dinucleotide, a well-known trigger of mutations in the human genome [16],[17]. Moreover changing an arginine residue to a stop codon seems to be a very common mutational mechanism in CHM, as described previously [13]. Deletion of exon 9 was found in three Spanish families sharing common haplotype, therefore suggesting a common ancestor. However, no identity by descent was observed in a Portuguese family carrying an additional and larger deletion involving also exon 9.

In our cohort we identified large rearrangements involving a translocation or complete deletions of *CHM*, as well as a wide range of out-of-frame partial deletions, nonsense and frameshift mutations. In all cases, these mutations, with the exception of the final exon-junction, presumably lead to the premature truncation of *CHM* mRNA. As exceptional cases, we have previously identified three different in-frame deletions (exon 2, exon 3 and 4, and exon 9 deletions) [6], with protein production detected. In these latter cases, the deletion involves a conserved protein domain implicated in the interaction with Rab proteins, crucial for the function of the REP-1 protein. As a result, due to REP-1 being completely absent, truncated or containing a dysfunctional domain, no functional protein is produced.

We cannot establish a reliable genotype-phenotype correlation in our cohort of patients, as has been widely reported for other cohorts [18]. Furthermore, we did not observe a correlation between the unprenylated Rab levels, measured in CHM patients´ fibroblasts, and their genotype as patients carrying the same mutation (a complete deletion of *CHM*) exhibited significantly different levels of unprenylated Rabs. Although these two siblings have a two-year age difference and thus it cannot be ruled out that unprenylated Rabs may accumulate over time leading to higher levels in the older sibling, these data further suggest that severity and progression of the disease may not be solely explained by the specific CHM mutation. The causes of underprenylation due to defects in the Rab prenylation machinery are complex and likely multifactorial [19]. However, it is known that REP2 can partially compensate a REP1 deficiency. Thus, a first area of investigation could be the analysis of REP2 levels to determine whether lower unprenylated Rab levels in some patients could be correlated with higher REP2 levels. A more complete study of prenylation status versus phenotype, as a factor of age, would be needed to determine whether unprenylated Rab levels could aid disease prognosis.

To conclude, this study identified six novel *CHM* mutations. Clinical history and the identification of molecular defects in *CHM* are not only important for current diagnostics and genetic counseling, but also for prenatal diagnosis and preimplantation genetic diagnosis. In addition, it is particularly relevant for guiding patient selection to be included in the outcome treatments.
